# Diversification of *CYCLOIDEA*-like genes in Dipsacaceae (Dipsacales): implications for the evolution of capitulum inflorescences

**DOI:** 10.1186/1471-2148-11-325

**Published:** 2011-11-06

**Authors:** Sara E Carlson, Dianella G Howarth, Michael J Donoghue

**Affiliations:** 1 Department of Ecology and Evolutionary Biology, Yale University, P.O. Box 208106, New Haven, Connecticut 06520-8106, USA; 2 Department of Biological Sciences, 8000 Utopia Pkwy, St John's University, Jamaica, New York 11439, USA; 3 Department of Ecology and Evolutionary Biology and the Peabody Museum of Natural History, Yale University, P.O. Box 208106, New Haven, Connecticut 06520-8106, USA; 4 Department of Evolutionary Botany, University of Neuchâtel, Rue Emile-Argand 11, CH-2009, Neuchâtel, Switzerland

## Abstract

**Background:**

*CYCLOIDEA (CYC)*-like genes have been implicated in the development of capitulum inflorescences (i.e. flowering heads) in Asteraceae, where many small flowers (florets) are packed tightly into an inflorescence that resembles a single flower. Several rounds of duplication of *CYC*-like genes have occurred in Asteraceae, and this is hypothesized to be correlated with the evolution of the capitulum, which in turn has been implicated in the evolutionary success of the group. We investigated the evolution of *CYC-*like genes in Dipsacaceae (Dipsacales), a plant clade in which capitulum inflorescences originated independently of Asteraceae. Two main inflorescence types are present in Dipsacaceae: (1) radiate species contain two kinds of floret within the flowering head (disk and ray), and (2) discoid species contain only disk florets. To test whether a dynamic pattern of gene duplication, similar to that documented in Asteraceae, is present in Dipsacaceae, and whether these patterns are correlated with different inflorescence types, we inferred a *CYC*-like gene phylogeny for Dipsacaceae based on representative species from the major lineages.

**Results:**

We recovered within Dipsacaceae the three major forms of *CYC*-like genes that have been found in most core eudicots, and identified several additional duplications within each of these clades. We found that the number of *CYC*-like genes in Dipsacaceae is similar to that reported for members of Asteraceae and that the same gene lineages (*CYC1*-like and *CYC2B*-like genes) have duplicated in a similar fashion independently in both groups. The number of *CYC*-like genes recovered for radiate versus discoid species differed, with discoid species having fewer copies of *CYC1*-like and *CYC2B*-like genes.

**Conclusions:**

*CYC*-like genes have undergone extensive duplication in Dipsacaceae, with radiate species having more copies than discoid species, suggesting a potential role for these genes in the evolution of disk and ray florets. The similarity in *CYC*-like gene diversification seen in Dipsacaceae and some members of the Asteraceae sets the stage to investigate whether the convergent evolution of capitulum inflorescences in both groups may have been underlain by convergent evolution in the same gene family.

## Background

Gene duplication is an important evolutionary force, providing the raw material for new genes that, if not lost, may be co-opted to perform novel functions [1,2]. Gene duplication has been implicated in the evolution of transcription factors that play key roles in developmental pathways and are likely involved in morphological evolution [3,4]. In plants, for example, the MADS-box and TCP gene families underwent duplication deep within angiosperm phylogeny, and these duplication events are thought to have played a role in major changes to flower morphology [5-12]. TCP genes are less well studied than MADS-box genes, but they are increasingly appreciated for their role in generating diverse floral forms.

The TCP gene family is named for the conserved helix-loop-helix (bHLH) TCP domain from TEOSINTE BRANCHED1 (*TB1*) in *Zea mays*, *CYCLOIDEA (CYC) *in *Antirrhinum majus*, and the proliferating cell factor (*PCF*) DNA-binding proteins in *Oryza sativa*. In *Arabidopsis*, the TCP gene family has 24 copies [13], which are divided into a clade of *PCF *genes, which control cell growth, and a clade of *CYC*/*TB1 *genes. These two clades differ in the length and sequence of the TCP domain, and some members of the *CYC*/*TB1 *clade have an additional conserved arginine rich "R domain" [14]. Within *CYC*/*TB1 *the "ECE" clade [15] contains a group of genes with a conserved short motif (glutamic acid-cysteine-glutamic acid) between the TCP and R domains. There are three major clades of ECE genes in the core eudicots, and the duplications leading to these copies predate this major lineage [10]. We refer to members of the ECE clade as "*CYC*-like" genes. The best studied of these genes, *CYC *itself, is required for the production of bilaterally symmetrical (zygomorphic, monosymmetric) flowers as a function of its expression in the dorsal portion of the floral meristem [16-18].

While *CYC-*like genes have been primarily studied in the context of floral symmetry in single flowers, they have also been implicated in the development of the capitulum (or "head") inflorescence in Asteraceae, a trait that may be associated with the evolutionary success of this group [19,20]. A typical capitulum consists of many small flowers (florets) packed tightly into a condensed head that can closely resemble a single large flower. In some groups, the florets in different parts of the capitulum are morphologically distinct. In radiate species, the outer florets (rays) are bilaterally symmetrical while the inner florets (disks) are radially symmetrical (actinomorphic, polysymmetric). In discoid species, there is little or no variation in floret morphology and the capitulum is composed of disk florets only. A third inflorescence type in Asteraceae, characteristic of the Lactuceae, consists of a capitulum with entirely bilaterally symmetrical flowers (ligulate florets). In Asteraceae, the evolution of *CYC*-like genes has been studied thoroughly in the radiate species *Helianthus annuus *(the cultivated sunflower), where ten copies have been recovered, which is more that in any other species to date [21]. Such extensive gene duplication is thought to be correlated with the evolution of ray versus disk florets [20,21]; however, this possible connection has not been investigated in any groups outside of the Asteraceae, nor has copy number been compared in clades with different inflorescence types.

**Figure 1:**
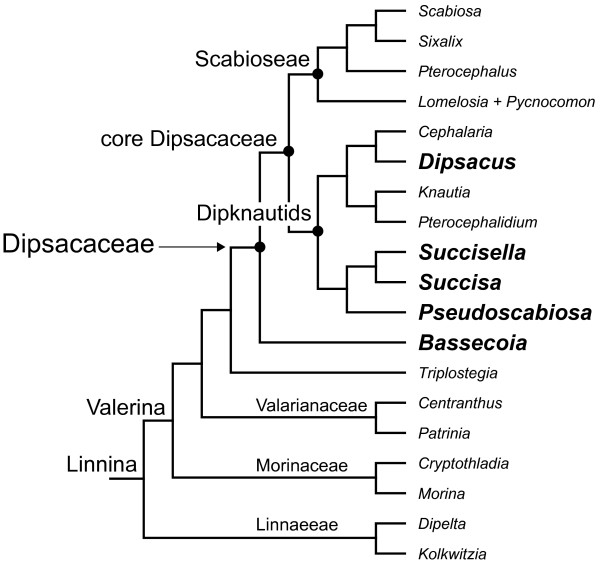
**Summary of phylogenetic relationships in Dipsacaceae**. Summary of major phylogenetic relationships in Dipsacaceae, with outgroups and clade names included. Species in larger bold font have discoid inflorescences.

Dipsacaceae (Dipsacales) is one of relatively few groups outside of Asteraceae where the capitulum inflorescence occurs and that also contains species with both radiate and discoid heads (although intermediates between floret types are present in radiate species and form a morphological continuum across the capitulum). Dipsacaceae is part of the Valerina clade of Dipsacales [22] and contains ca. 300 species of perennial and annual herbs that are divided into three main clades [23]: *Bassecoia *(3 spp.), Scabioseae (ca. 150 spp.), and the Dipknautid clade (ca. 150 spp.), the latter two of which comprise the "core Dipsacaceae" (Figure 1). Most Dipsacaceae species have radiate capitula, with the exception of a few small lineages. These include *Bassecoia*, which is sister to the remaining Dipsacaceae, and two groups within the Dipknautid clade: *Dipsacus *(ca. 20 species) and a small clade composed of *Succisa *(three species), *Succisella *(five species), and *Pseudoscabiosa *(three species). Howarth and Donoghue [15,24] investigated the evolution of *CYC*-like and *DIVARICATA*-like (*DIV*; a MYB gene family also involved in the floral symmetry pathway [25]) genes in Dipsacales, and found additional duplications of *CYC*-like genes in *Dipsacus pilosus*, one of two representatives of Dipsacaceae included in the analysis, as well as additional duplications of *DIV*-like genes in *Sixalix atropurpurea*, the sole representative of Dipsacaceae included in the study of *DIV*-like genes. Given the unusual floral characteristics associated with this group (e.g., the presence of a capitulum and a sometimes zygomorphic epicalyx [26]), further investigation of *CYC*-like genes from a broader sampling of Dipsacaceae is warranted.

The aim of this study is to investigate patterns of gene duplication in *CYC*-like genes and relate patterns of gene evolution to the evolution of radiate versus discoid inflorescences. We also seek to compare the diversification of *CYC*-like genes in Dipsacaceae to that reported for members of Asteraceae (e.g. *Helianthus*), to test for a similar pattern of gene duplication. This study represents a first step in eventually deciphering the involvement of *CYC*-like genes in determining capitulum form and differences in floral symmetry in Dipsacaceae.

## Results

### Phylogenetic Analysis

**Figure 2:**
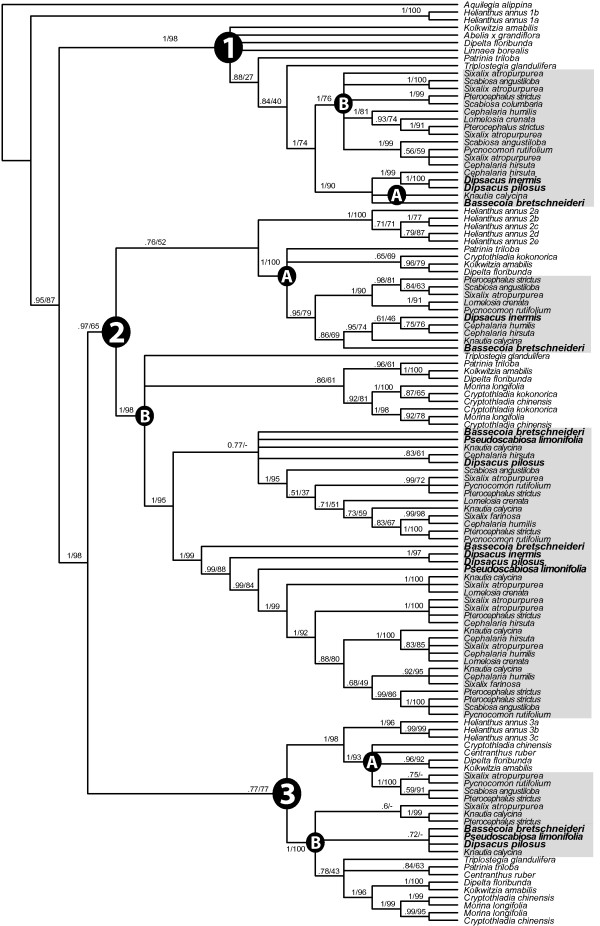
***CYC*-like gene phylogeny in Dipsacaceae**. Majority-rule consensus tree of *CYC-*like genes with the major clades (1-3) and subclades (A, B) indicated. Numbers above branches are support values (Bayesian posterior probabilities/maximum likelihood bootstrap values) and members of Dipsacaceae are shaded in grey. Species names in larger bold font have discoid inflorescences.

**Figure 3:**
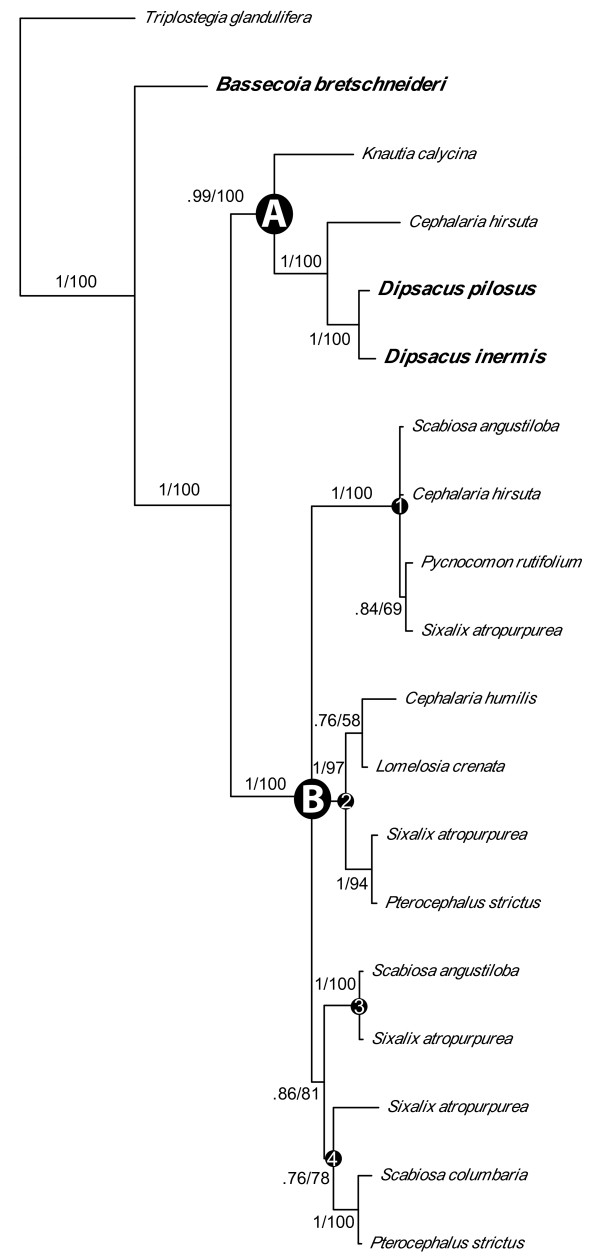
**Phylogenetic relationships in *Dpc*CYC1**. Majority-rule consensus tree of *DpcCYC1 *with the major clades (A and B) and putative copies (1-4) indicated. Numbers above branches are support values (Bayesian posterior probabilities/maximum likelihood bootstrap values). Species names in larger bold font have discoid inflorescences.

**Figure 4:**
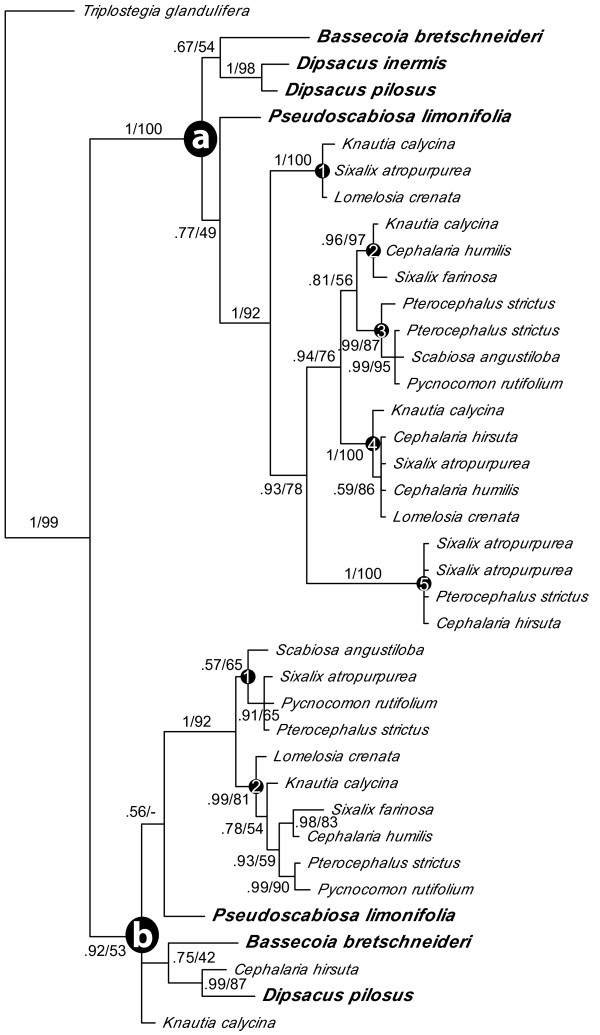
**Phylogenetic relationships in *DpcCYC2B***. Majority-rule consensus tree of *DpcCYC2B *with the major clades (A and B) and additional putative copies (1-5) indicated. Numbers above branches are support values (Bayesian posterior probabilities/maximum likelihood bootstrap). Species names in larger bold font have discoid inflorescences.

The combined matrix contains 121 sequences and 474 bases. Alignment of the TCP and R domains is unambiguous across taxa, however, the intervening region is difficult to align; therefore, we omitted ambiguous portions of this region from the alignment. All datasets, as well as the full-length alignments, are available in TreeBASE http://www.treebase.org or upon request from the first author. To improve phylogenetic resolution within *DpcCYC1 *and *DpcCYC2B*, we analyzed these sequences separately in order to align the intervening region between the TCP and R domain. The aligned length of the *DpcCYC1 *matrix was 369 bases with 19 sequences and the *DpcCYC2B *matrix was 231 bases with 39 sequences. The tree topologies of the Bayesian and maximum likelihood (ML) analyses were congruent with only minor differences in branch lengths, and consensus trees are shown in Figures 2, 3, 4.

We recovered between 2 and 12 copies for each species. While we extensively screened 14 representatives of the major lineages of Dipsacaceae, we did not recover all hypothesized copies from each species, which may have been the result of unsuccessful amplification or absence/loss from those genomes. Our analysis recovered the three main clades of *CYC*-like genes that have been reported for core eudicots and the additional duplications previously found in representatives of Dipsacaceae (i.e. *DipsCYC1*, *DipsCYC2Ba*, *DipsCYC2Bb*, and *DipsCYC3B) *[15]. We name the three major clades of Dipsacaceae *CYC*-like genes: *DpcCYC1*, *DpcCYC2*, and *DpcCYC3*. Also consistent with previous results is the sister relationship of *DpcCYC2 *and *DpcCYC3*. Novel findings include copies *DpcCYC2A *and *DpcCYC3A*, whose orthologs had been inferred for other members of the Dipsacales [15], and additional duplications within *DpcCYC1 *(Figure 3) and within *DpcCYC2Ba *and *DpcCYC2Bb *(Figure 4), which are unique to Dipsacaceae. The additional duplications within these clades are found only in species with radiate capitula.

*DpcCYC1 *is well supported, with the sequence from *B. bretschneideri *resolved as sister to two clades of *DpcCYC1 *in the separate analysis (*DpcCYC1A *and *DpcCYC1B*; Figure 3). That is, the duplication within *DpcCYC1 *appears to have occurred after the core Dipsacaceae split from *Bassecoia. DpcCYC1A *is recovered only from members of the Dipknautid clade, including both discoid (i.e. *D. pilosus*) and radiate (i.e. *C. hirsuta*, *K. calycina*) species, while *DpcCYC1B *is found only in radiate species from Scabioseae and the Dipknautids. Within *DpcCYC1B*, there are four clades ("*1B1*-*1B4*") representing four putative copies (although Bayesian support for *1B4 *was low: ML bootstrap = 78, posterior probability = 0.76).

The *DpcCYC2 *clade is the largest and most complicated group in terms of duplication events and this clade aligns with five copies of *Helianthus*, which has undergone a similar radiation in this gene lineage. We found evidence for one copy of *DpcCYC2A *in all major lineages of Dipsacaceae, and within this clade, the gene phylogeny is generally consistent with the species phylogeny [23]. With the separate analysis of *DpcCYC2B*, we were able to further clarify relationships within this gene lineage (Figure 4). The two major copies that were previously reported [15] - *DpcCYC2Ba *and *DpcCYC2Bb *- are hereafter referred to as "*2Ba*" and "*2Bb*" for simplicity. In the combined analysis of all *CYC*-like genes, the *DpcCYC2Ba *sequence from *B. bretschneideri *is sister to sequences from all remaining species in this clade, similar to the placement of the sequences from *B. bretschneideri *in *DpcCYC1*. In the separate analysis of *DpcCYC2Ba*, however, the position of the sequence from *B. bretschneideri *is unresolved but joins sequences from the other discoid species *Pseudoscabiosa limonifolia*, *Dipsacus pilosus*, and *Dipsacus inermis *outside a large clade composed only of sequences from radiate species from both the Scabioseae and Dipknautid groups. We inferred five putative copies within this core clade of *2Ba *(*2Ba1 *- *2Ba5*; Figure 4). Similarly, within the *2Bb *lineage, we found only one copy in the discoid species (i.e. *B. bretschneideri*, *P. limonifolia*, *D. pilosus*). Sequences from these species, along with the Dipknautid radiate species *Knautia calycina *and *Cephalaria hirsuta*, are resolved outside of a clade composed of sequences from radiate species from both the Dipknautids and Scabioseae. Within this core clade of *2Bb*, we inferred two putative copies (*2Bb1*, *2Bb2*), although support for *2Bb1 *was low (i.e. ML bootstrap = 65, posterior probability = 0.57).

Lastly, we recovered the fewest sequences from the *DpcCYC3 *clade. Within *DpcCYC3*, we found evidence for one copy of *DpcCYC3A *for members of Scabioseae, but did not recover this gene for *Bassecoia *or members of the Dipknautid clade. *DpcCYC3B *was recovered for four species that represent the three major lineages of Dipsacaceae (*Bassecoia*; *Pseudoscabiosa *and *Knautia *from the Dipknautid clade; *Sixalix *from Scabioseae) and there were two copies of this gene for *K. calycina*.

### Sequence characteristics

**Figure 5:**
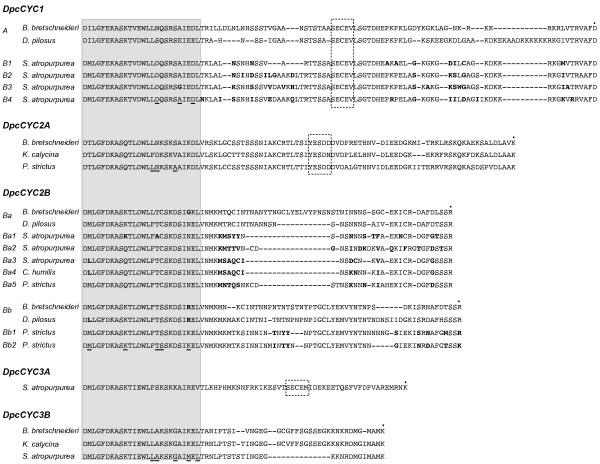
**Protein alignment**. Protein alignment of representative species of the TCP domain, intervening region, and R domain for the major *DpcCYC *copies aligned independently of one another. The TCP domain is boxed and shaded, the "ECE" region is shown in a dashed box, and the start of the R domain is indicated with a dot. Bases that are in bold represent polymorphisms between putative copies within *DpcCYC1B, DpcCYC2Ba*, and *DpcCYC2Bb*, and underlined bases represent fixed differences in the TCP domain within the five major copies.

The three major clades of *DpcCYC*, as well as the putative copies within these clades, differ in sequence characteristics, most notably the length between the TCP and R domain (Figure 5). *DpcCYC1 *is the longest copy, with fewer gaps in the intervening region (suggesting these sequences may be less diverged from each other than the other copies), followed by *DpcCYC2A*. Duplications within both *DpcCYC2 *and *DpcCYC3 *appear to have been accompanied by a reduction in sequence length between the A and B copies, and the B copies of *DpcCYC2 *and *DpcCYC3 *have the shortest sequence lengths. The "ECE" region, the characteristic conserved domain found in the intervening region, is easily identifiable in *DpcCYC1*, *DpcCYC2A*, and *DpcCYC3A*, but apparently absent in all copies of both *DpcCYC2B *and *DpcCYC3B *(the ECE region is also missing from these genes in other members of the Dipsacales examined [15]). In addition to differences in the intervening region, there are also amino acid differences in the TCP domain between the different copies. *DpcCYC1 *is the most divergent from the other copies. There are also notable differences in sequence characteristics between the putative copies within each of the subclades, including the length of the intervening region as well as amino acid polymorphisms.

## Discussion

Our study verifies that gene duplication events yielded five major copies of *CYC*-like genes prior to the origin of Dipsacaceae (*DpcCYC1*, *DpcCYC2A*, *DpcCYC2B*, *DpcCYC3A*, and *DpcCYC3B*) [15]. The major duplication found in *DpcCYC1 *(*DpcCYC1A*, *DpcCYC1B*) and the additional putative copies within *DpcCYC1B *are found in Dipsacaceae but not in other Dipsacales. Similarly, the major duplication in *DpcCYC2B *(*2Ba*, *2Bb*) is consistent with previous studies [15], but the additional duplications within these copies are unique to Dipsacaceae. These duplications do not appear to be the result of recent polyploidization events, as they do not occur across all copies. Additionally, chromosome counts indicate that there is no evidence for genome doubling in the species used in this study [27-29] (although, ancient whole genome duplication in the ancestor of Dipsacaceae has not been investigated). We feel that the newly discovered subclades within *DpcCYC1 *and *DpcCYC2B *likely represent different copies, as opposed to alleles, because they contain species from throughout the Dipsacaceae phylogeny. In other words, we would not expect allele sequences to be conserved across distant relatives.

### Gene trees in relation to the species tree

Congruence between gene trees and the species tree within Dipsacaceae is difficult to interpret. Outgroups in the Linnina clade are generally resolved as sister to Dipsacaceae, although phylogenetic relationships between these species tend to be poorly supported. The two *Helianthus CYC1*-like copies are resolved outside of the *CYC1*-like clade, indicating that better phylogenetic sampling is needed to clarify relationships among copies in this part of the tree. Within *DpcCYC1*, the failure to amplify *DpcCYC1A *from members of Scabioseae (or the loss of this copy from these genomes), combined with the additional copies in *DpcCYC1B*, renders species relationships within this clade unclear. The *DpcCYC2A *tree (with no duplications) is more or less consistent with phylogenetic studies [23,30,31], although the position of *B. bretschneideri *is weakly resolved (i.e. < .90 posterior probability, < 70% ML bootstrap support) with members of the Dipknautids rather than as sister to all other Dipsacaceae, as in the species phylogeny. Within *DpcCYC2B*, the presence of multiple copies precludes the inference of species relationships. Lastly, congruence between the gene trees and the species tree in both copies of *DpcCYC3 *cannot be determined due to limited taxon sampling and poor phylogenetic signal in the gene trees (likely the result of short sequence length). Based on these results, the utility of most *DpcCYC *genes for use in phylogenetic studies would be problematical due to the presence of multiple putative copies, low amplification success for some genes (e.g. the *DpcCYC3*'s), short sequences with a highly conserved TCP domain that yield low phylogenetic signal, and difficulties in aligning the intervening region. A possible exception is *DpcCYC2A*, which shows some promise in elucidating species relationships. This gene may be enhanced as a phylogenetic marker by using the full-length sequence rather than the shorter region between the TCP and R domain used in this study.

Strikingly, we found no additional duplications in any of the major subclades for *Bassecoia bretschneideri*. In the separate analysis of *DpcCYC1*, the main duplication event occurred after *Bassecoia *split from the core Dipsacaceae. Similarly, we found no evidence for additional duplications in *DpcCYC1 *in *Dipsacus inermis, Dipsacus pilosus*, or *Pseudoscabiosa limonifolia*, the other discoid species included in this study. In the *2Ba *and *2Bb *clades, both *Dipsacus *species and *P. limonifolia *occupy a position along with *B. bretschneideri *(plus *Knautia calycina *and *Cephalaria hirsuta *in *2Bb*) outside of the core clades of these lineages that include all radiate species. Given these results and our current understanding of Dipsacaceae phylogeny (Figure 1[23]), it is perhaps most likely that the *2Ba *and *2Bb *genes duplicated in the lineage leading to the core Dipsacaceae and were subsequently lost twice: once in the lineage leading to *Pseudoscabiosa *and once in *Dipsacus*. However, we cannot rule out that by using a degenerate primer approach we failed to amplify these copies in *Bassecoia*, *Pseudoscabiosa*, and *Dipsacus*. In any case, the occurrence of the same general pattern of duplication in three different *CYC*-like genes (*DpcCYC1, DpcCYC2Ba*, and *DpcCYC2Bb*) is potentially of great interest.

### Diversification of *CYC*-like genes and character evolution

**Figure 6:**
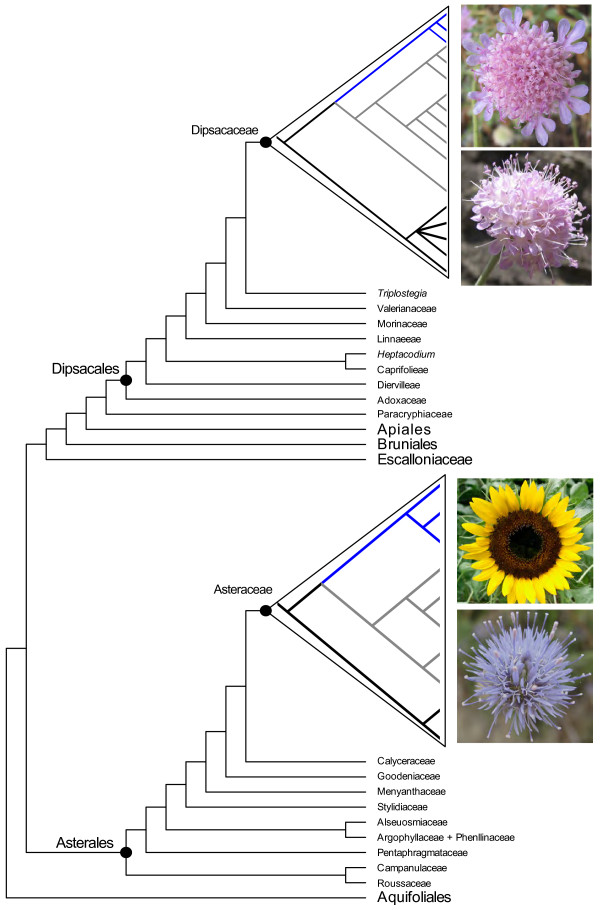
**Summary Campanulidae phylogeny with plotted *CYC*-like gene trees**. Summary of phylogenetic relationships within the Campanulideae following Tank and Donoghue [45] with *CYC*-like gene tree for *Helianthus annus *[21] plotted within the Asteraceae clade and *CYC*-like gene tree for Dipsacaceae plotted within the Dipsacaceae clade (clades are color coded: black = *CYC1*, grey = *CYC2*, blue = *CYC3*). The radiate *Lomelosia cretica *(top photo) and the discoid *Bassecoia bretschneideri *(bottom photo) provide examples of inflorescence form in Dipsacaceae. The radiate *Helianthus annuus *(top photo) and the discoid *Echinops sp*. (bottom photo) provide examples of inflorescence form in Asteraceae.

The pattern of *CYC*-like gene diversification appears to generally correlate with inflorescence form in Dipsacaceae. Groups with radiate capitula are represented in the major clades and subclades of *DpcCYC*, while the discoid groups *Bassecoia*, *Pseudoscabiosa*, and *Dipsacus *appear to have only one copy of *DpcCYC1 *(or no copy in the case of *Pseudoscabiosa*), *2Ba*, and *2Bb*. While *CYC*-like genes have been investigated in functional studies of other Asteraceae species (e.g. *Gerbera *with one copy of *CY1*-like and three copies of *CYC2*-like genes, and *Senecio *with two copies in the *CYC2*-like clade [19,20]), gene duplication has been investigated most extensively in *Helianthus*. The highest reported number of *CYC*-like genes is 10 for *Helianthus annuus *[21], a radiate species of Asteraceae, and the pattern of diversification in radiate Dipsacaceae groups is remarkably similar. While we did not recover all copies for each species, we hypothesize that there are at least 15 and possibly 17 copies of *CYC*-like genes in radiate members of Dipsacaceae (Figure 6), depending on better resolution within *2Bb *and whether the duplication in *DpcCYC3B *found in *Knautia *can be generalized to other radiate species. For discoid groups, our study infers 5 copies.

The duplications in *Helianthus *and radiate Dipsacaceae occur within the same gene lineages. *Helianthus *has two copies of *CYC1*-like genes and we recovered five putative copies for radiate species of Dipsacaceae. Most notably, there are five copies of *Helianthus *in the *CYC2*-like clade, and seven or eight (depending on resolution in *2Bb*) copies in radiate species of Dipsacaceae. Lastly, *Helianthus *has three copies in the *CYC3*-like clade, while members of Dipsacaceae appear to have at least two (possibly three in *Knautia*), although copy number was difficult to assess for this gene clade due to problems with amplification. If we assume that most *CYC*-like genes that are present have been discovered, then it is possible that the independent evolution of radiate capitula in Asteraceae and Dipsacaceae may have been associated with independent duplication events in orthologous *CYC*-like genes. However, in the absence of expression data for *CYC*-like genes in Dipsacaceae and broader studies of gene duplication events within Asteraceae, this is clearly speculative. To date, the evolutionary history of *CYC*-like genes has not been investigated in discoid Asteraceae (or Asteraceae with inflorescences composed of ligulate florets), so it is unknown whether these species also diverged in copy number from their radiate relatives.

The additional duplications in *CYC1-*like and *CYC2B*-like genes are of particular interest because, within the core eudicots, they may be specific to *Helianthus *and Dipsacaceae, at least within the species that have been surveyed thus far (outside of the core eudicots, two copies of *CYC1*-like genes are found in Papaveraceae, [32]). In most previous studies, only a single copy of *CYC1*-like genes has been found [33,10]. Our finding that only members of the Dipknautid clade have *DpcCYC1A *is potentially interesting given the differences in floral traits between the main clades of Dipsacaceae. Members of Scabioseae have five petals while the Dipknautids (and *Bassecoia*) have four, and the epicalyx in Scabioseae is characterized by several structural modifications that are not present in *Bassecoia *or in the Dipknautids [34]. However, functional information for *CYC1*-like genes is limited, although putative orthologs in *Arabidopsis *are thought to be involved in branching architecture [35]. *TB1 *is similar in sequence to genes in the *CYC1*-like clade, and is also responsible for differences in branching architecture between cultivated maize and its wild progenitor, teosinte [36]. While there are no functional data for *Helianthus*, the two copies of *CYC1*-like genes (*HaCYC1a *and *HaCYC1b*) were found to diverge in expression patterns, with *HaCYC1a *expressed across all tissues except roots, and *HaCYC1b *expressed only in the petals [21]. Due to potential differences in *CYC1*-like genes between Scabioseae and the Dipknautid clade and the presence of multiple copies of *DpcCYC1B *in radiate species, *CYC1*-like genes are attractive candidates for future study.

Previous studies have generally found more duplications in the *CYC2*-like genes than in the *CYC1*-like or *CYC3*-like genes; however, no more than three copies have been reported outside of *Helianthus *and Dipsacaceae [10]. The *CYC2*-like clade includes *CYC *from *Antirrhinum*, and its orthologs have been studied in several plant groups, including in members of Asteraceae, where it has been suggested that *CYC*-like genes might be responsible for differentiating the radially symmetrical disk florets from the bilaterally symmetrical ray florets [37]. In both *Gerbera *and *Senecio*, *CYC2-*like genes are expressed in ray florets and functional data suggest they are involved in differentiating the radially symmetrical disk florets from the bilaterally symmetrical ray florets [19,20]. While no functional data exist for the *Helianthus *orthologs of *CYC2*-like genes, their expression patterns were found to diverge, which may be indicative of sub and/or neofunctionalization [38] of the duplicated *CYC2*-like copies. While our correlational study cannot establish causation, the higher number of *DpcCYC2B *genes in radiate versus discoid Dipsacaceae suggests that they might also play a role in the development of ray florets. Expression studies and functional data are needed to address this possibility.

## Conclusions

The results presented here represent the first step in assessing the role of *CYC*-like genes in Dipsacaceae development. We recovered the five major clades that were reported in previous studies and discovered several additional copies, most notably in the *DpcCYC1 *and *DpcCYC2B *clades. The *DpcCYC2B *clade in particular demonstrates a dynamic history of gene duplication. Interestingly, the pattern of *CYC*-like diversification in Dipsacaceae is very similar to that of *Helianthus annuus*, a species of Asteraceae that has been screened for *CYC*-like genes. Our study also suggests that the number of *DpcCYC *copies tends to correlate with the distribution of radiate versus discoid inflorescence form, with species bearing radiate heads having more than twice the number of *CYC*-like genes as discoid species. Investigations of *CYC*-like genes in discoid members of Asteraceae are needed to assess the generality of this pattern.

Capitulum inflorescences have evolved independently in a number of angiosperm lineages, but perhaps most often within the Campanulidae, a clade of over 30,000 species that includes both the Asteraceae and the Dipsacaceae. Many campanulids produce flat-toped inflorescences containing many small flowers, and this arrangement appears to have set the stage both for the differentiation of enlarged (often sterile and bilaterally symmetrical) flowers around the periphery of the inflorescence (e.g., *Viburnum *in the Adoxaceae) and for the condensation of the inflorescence into a capitulum (e.g., in Asteraceae and its sister group Calyceraceae; Dipsacaceae; *Eryngium *and others within Apiaceae). Such inflorescences could have originated in each of these lineages via different pathways, deploying different sets of genes. Instead, our analyses indicate that two widely separated groups within the campanulid angiosperms - Asteraceae and Dipsacaceae - may have taken advantage of gene duplications within the same family of genes.

## Methods

### Plant material

Fourteen individuals were studied, representing all major subclades of Dipsacaceae, from either herbarium specimens or silica-preserved field collections. Total genomic DNA was extracted using Qiagen DNEasy methods (Qiagen, Valencia, California), or a modified version using beta-Mercaptoethanol and proteinase-K for herbarium specimens [39]. We also included 44 published sequences of outgroups from the Linnina clade [22] and all ten *CYC-*like genes published for *Helianthus annuus*. We used a single copy from *Aquilegia *(Ranunculaceae) as the outgroup [10]. All taxa used in this study are listed in additional file 1.

### Polymerase chain reaction and sequencing

Degenerate primers designed by Howarth and Donoghue [10] were first used to amplify the TCP domain (forward primer) and the R domain (reverse primer). Amplification with these primers used the following cycling program: 95°C for 45 s, 50°-56°C for 1 min, and 72°C for 1 min 30 s, repeated for 39 cycles. Reactions were performed using Taq DNA polymerase (QIAGEN, Valencia, CA) in 25 μL, with final concentrations of 2.5 mM MgCl_2_, 0.5 mM of each primer, 0.8 mM dNTPs, and 0.5× Q Solution (QIAGEN). Amplified products were cloned using the Invitrogen TOPO TA cloning kit for sequencing (Invitrogen, Carlsbad, CA). Between 25 and 100 (depending on cloning success) colonies were screened for all potentially different copies of *CYC*-like genes. Colonies were picked and mixed directly into a PCR cocktail (in the same concentrations as above with standard M13 primers). Following a 10 minute denaturing step at 95°C, the following program was used: 95°C for 30 s, 55°C for 45 s, and 72°C for 60 s, repeated for 24 cycles. Cloned PCR products between 200-800 bp were cleaned as above. Sequences were generated using dye terminator cycle sequencing using ABI PRISM "Big Dye" Primer Cycle Sequencing Ready Reaction kits (Perkin-Elmer, Foster City, California), and visualized using an ABI3730 (Applied Biosystems DNA Analyzer). Through the course of the study, we designed additional primers specific to Dipsacaceae (Table 1).

**Table 1:** Primer sequences.

*CYC *copy	Name	5' to 3'
**Forward primer**		

*CYC1*	CYC1F	GACATKCTBGGATTCGARAAGGC

*CYC2A*	CYC2AF	AAAGATTTGGTMMDRTCCAATCT

*CYC2Ba*	CYC2BaF	GGTATACCCCATTTTCACATCTTAC

*CYC2Bb*	CYC2BbF	GTATACCCCATTTTCACATCTTACATCA

*CYC3A*	CYC3A	ATACATATGTATCCTTTAGCCAATACA

*CYC3B*	CYC3BF	GAAACATTTACCTGCAACCCA

**Reverse primer**		

*CYC1*	CYC1R	GCTCTTTCTCTYGCYTTYGCCCT

*CYC2A*	CYC2AR	CGYGTGTTGYTACCGATCTCC

*CYC2Ba *&*CYC2Bb*	CYC2R	TCTTGCTCTTTCYCTYGCYTTYGCCCTA

*CYC3A *&*CYC3B*	CYC3R	TGCTCTTTCTCTCGCYTTCGCCCTAGC

Primer sequences used in this study.

### Phylogenetic alignment and analysis

All clones were assembled in Sequencher (Gene Codes Corp., Ann Arbor, Michigan) and identified as *CYC*-like genes based on the conserved amino acid sequence of the TCP domain. Clones were assembled into different sequence "groups" according to shared differences. Members of the same sequence group were generally identical and only varied by obvious polymerase error (single base differences in one or two clones out of several, with different clones being mutated at different sites). A consensus sequence was generated for each group and exported for phylogenetic analysis. We analyzed two different datasets: one with all *CYC*-like sequences included, and two additional dataset with only members of *DpcCYC1 *or *DpcCYC2B*. We analyzed these clades separately in order to align more of the intervening region to aid phylogenetic resolution.

Aligned datasets were generated using Muscle [40] and adjusted manually in MacClade v. 4.06 [41]. Models of molecular evolution were calculated using AIC (Akaike Information Criteria) scores in ModelTest version 3.7 [42] (model = GTR + G) and implemented in the Bayesian analyses. Bayesian inference analyses were performed using MrBayes version 3.1.2 [43] and two simultaneous runs were initiated from random starting trees. Posterior probabilities of trees were approximated using the Metropolis-coupled Markov chain Monte Carlo (MC3) algorithm with four incrementally heated chains (T = 0.2) for 20 million generations and sampling trees every 2,000 generations. Convergence and sampling intensity were evaluated using PRSF (all parameters approached 1.0) and ESS (values were greater than 200). The average standard deviation of split frequencies approached 0.01 in all analyses, indicating that each MC3 chain converged to the target distribution. To estimate burn-ins, posterior parameter distributions were viewed using Tracer version 1.4.

ML analyses were conducted using RAxML version 7.0.4 [44]. Tree searches were executed from a random stepwise-addition maximum parsimony tree and employed the GTRGAMMA nucleotide substitution model. ML analyses were run ten times per dataset, starting from ten different starting trees. All free model parameters were estimated with RAxML, with GAMMA model parameters estimated up to an accuracy of 0.1 log likelihood units. Nonparametric bootstrapping under ML was also carried out with RAxML using 1,000 bootstrap replicates.

## Authors' contributions

All authors contributed to the design of the study. SEC collected the plant material (travelled to herbaria, conducted fieldwork) and performed the lab work and analysis with input from DGH. SEC wrote a first draft with contributions from DGH and MJD. All authors read and approved the final manuscript.

## Supplementary Material

Additional file 1 **Species included in this study**. Species used in this study, with voucher information and GenBank numbers. PDF document. Click here for file
