# Supplementary material for: Diversification of CYCLOIDEA-like genes in Dipsacaceae (Dipsacales): implications for the evolution of capitulum inflorescences
Source: BMC Evol Biol. 2011 Nov 6;11:325. doi: 10.1186/1471-2148-11-325 (PMC3224765; doi:10.1186/1471-2148-11-325)
Supplement: Additional file 1 — Species included in this study. Species used in this study, with voucher information and GenBank numbers. PDF document. [file 1471-2148-11-325-S1.PDF]

**Species**	**Voucher**	***CYC*-like Genes:**								
		**1**	**1A**	**1B**	**2A**	**2B**	**2Ba**	**2Bb**	**3A**	**3B**
Outgroups										
Aqulegia alippina		DQ462258								
Helianthus annuus		EU088366<br>EU088367				EU088368<br>EU088369<br>EU088370<br>EU088371<br>EU088372			EU088373<br>EU088374<br>EU088375	
Abelia x grandiflora	*Donoghue, voucher lacking*	AY851165								
Cryptothladia chinensis	*Boufford et al 27870* (A)				AY851189	AY851187<br>AY851188			AY851226	AY851227,<br>AY851228
Centranthus ruber	*Bell 203* (YU)								AY851224	AY851225
Dipelta floribunda	*Buckland and Kelly 32* (A)	AY851167			AY851194	AY851195			AY851232	AY851233
Kolkwitzia amabilis	*Elsik et al 844* (A)	AY851170			AY851199	AY851200			AY851237	AY851238
Linnea borealis	*Donoghue 113, voucher lacking*	AY851172								
Morina longifolia	*Eriksson s.n. 2 Nov. 1999* (SBT)					AY851205<br>AY851206				AY851244,<br>AY851245
Patrinia triloba	*Eriksson 807* (SBT)	AY851175			AY851207	AY851208				AY851246
Triplostegia glandulifera	*Boufford et al 27738* (A)	AY851181				AY851213				AY851252
Dipsacaceae										
Bassecoia bretschneideri	*Boufford et al. 35478* (A)	JN944774			JN944790		JN944800	JN944822		JN944845
Pseudoscabiosa limonifolia	*Schimmenti & Scafidi s.n.* (MRSN)						JN944801	JN944823		JN944844
Dipsacus inermis	*Edinburgh Nepal Expedition (2001)* (E)		JN944775		JN944791		JN944802			
Dipsacus pilosus	*No voucher*		AY851168				AY851198	AY851197		AY851234
Cephalaria hirsuta	*Holtz et al. 779* (E)		JN944776	JN944778	JN944792		JN944814<br>JN944818	JN944824		
Cephalaria humilis	*Hilliard & Burtt 18859* (E)			JN944782	JN944793		JN944807<br>JN944816	JN944831		
Knautia calycina	*Cellinese 6000* (YU)		JN944777		JN944794		JN944803<br>JN944806<br>JN944813	JN944825<br>JN944832		JN944842<br>JN944843
Scabiosa angustiloba	*Hilliard and Burtt 10838* (E)			JN944779<br>JN944786	JN944795		JN944811	JN944826	JN944836	
Scabiosa columbaria	*Bell 199*, voucher lacking			AY851177						

**Species**	**Voucher**	***CYC*-like Genes:**								
		**1**	**1A**	**1B**	**2A**	**2B**	**2Ba**	**2Bb**	**3A**	**3B**
Sixalix atropurpurea	*Carlson 137* (YU)			JN944780<br>JN944783<br>JN944787<br>JN944788	JN944796		JN944804<br>JN944815<br>JN944819<br>JN944820	JN944827	JN944837	JN944840
Sixalix farinosa	*No voucher*						JN944808	JN944835		
Pterocephalus strictus	*Archibald 8316* (E)			JN944785<br>JN944789	JN944797		JN944809<br>JN944810<br>JN944821	JN944828<br>JN944833	JN944838	JN944841
Pycnocomon rutifolium	*Sales & Hedge 02/16* (E)			JN944781	JN944798		JN944812	JN944829<br>JN944834	JN944839	
Lomelosia crenata	*Archibald 443* (E)			JN944784	JN944799		JN944805<br>JN944817	JN944830		
